# Robust Detection and Genotyping of Single Feature Polymorphisms from Gene Expression Data

**DOI:** 10.1371/journal.pcbi.1000317

**Published:** 2009-03-13

**Authors:** Minghui Wang, Xiaohua Hu, Gang Li, Lindsey J. Leach, Elena Potokina, Arnis Druka, Robbie Waugh, Michael J. Kearsey, Zewei Luo

**Affiliations:** 1 Laboratory of Population & Quantitative Genetics, The State Key Laboratory of Genetic Engineering, Institute of Biostatistics, School of Life Sciences, Fudan University, Shanghai, China; 2 School of Biosciences, The University of Birmingham, Edgbaston, Birmingham, United Kingdom; 3 Scottish Crop Research Institute, Invergowrie, Dundee, United Kingdom; Washington University, United States of America

## Abstract

It is well known that Affymetrix microarrays are widely used to predict genome-wide gene expression and genome-wide genetic polymorphisms from RNA and genomic DNA hybridization experiments, respectively. It has recently been proposed to integrate the two predictions by use of RNA microarray data only. Although the ability to detect single feature polymorphisms (SFPs) from RNA microarray data has many practical implications for genome study in both sequenced and unsequenced species, it raises enormous challenges for statistical modelling and analysis of microarray gene expression data for this objective. Several methods are proposed to predict SFPs from the gene expression profile. However, their performance is highly vulnerable to differential expression of genes. The SFPs thus predicted are eventually a reflection of differentially expressed genes rather than genuine sequence polymorphisms. To address the problem, we developed a novel statistical method to separate the binding affinity between a transcript and its targeting probe and the parameter measuring transcript abundance from perfect-match hybridization values of Affymetrix gene expression data. We implemented a Bayesian approach to detect SFPs and to genotype a segregating population at the detected SFPs. Based on analysis of three Affymetrix microarray datasets, we demonstrated that the present method confers a significantly improved robustness and accuracy in detecting the SFPs that carry genuine sequence polymorphisms when compared to its rivals in the literature. The method developed in this paper will provide experimental genomicists with advanced analytical tools for appropriate and efficient analysis of their microarray experiments and biostatisticians with insightful interpretation of Affymetrix microarray data.

## Author Summary

One of the ultimate goals of genomics is to explore structural and functional variations of all genes in a genome. High-density oligo-microarray techniques enable prediction of genome-wide gene expression and genome-wide genetic polymorphisms from using RNA and genomic DNA samples, respectively. A recent proposal to integrate the two predictions by use of RNA microarray data alone has great practical implications in genomics. However, it is essential but very challenging to develop an appropriate analytical method for detecting genetic polymorphisms (SFPs) from RNA expression data, which are inherently coupled with various sources of biological and technical variations. This paper presents a novel statistical approach to detect SFPs from gene expression data. We demonstrated that the new method is significantly more robust to variation due to differential expression of genes and improves the reliability of calling SFPs that bear genuine sequence polymorphisms than the other five methods in the mainstream literature on SFP prediction from microarray data. The improved predictability of detecting SFPs not only confers accuracy in evaluating gene expression from microarray information, but also opens up an opportunity to integrate structural and functional analyses by using only one set of microarray data.

## Introduction

Microarray technology has stimulated tremendous research interest in exploring genetic polymorphisms and gene expression at the genome wide level. Several studies have reported the use of oligonucleotide arrays for sequence variation discovery in a highly parallel manner. Winzeler et al pioneered the development of a high-throughput genotyping platform by hybridizing labelled total genomic DNA to oligonucleotide arrays [1]. In yeast, it has proven useful in linkage analysis, the dissection of quantitative trait loci, and in assessing species population structure [2]. Recently, this approach has been applied to organisms with more complex genomes, such as *Arabidopsis thaliana* [3], to assess the molecular basis of natural phenotypic variation. This type of sequence variation detected by a single probe in an oligonucleotide array was termed a *Single-Feature Polymorphism* (SFP), where a feature refers to a probe in the array [3]. By hybridizing cRNA from two parental yeast strains and their segregants onto yeast Affymetrix GeneChip arrays, Ronald et al was probably the first to propose the concept of simultaneous genotyping and gene expression analysis, and developed a method for identifying SFPs and genotyping the yeast strains at the SFPs mainly by combining a *k*-means clustering analysis and a mixture population analysis [4]. The idea behind the approach was the proposition that the presence of polymorphism in a perfect-match (PM) probe sequence between the parental strains, one of which was assumed to have the same reference sequence as the probe sequence, would lead to a detectable difference between the observed PM value of the probe and its corresponding predicted value from the positional-dependent-nearest-neighbour (PDNN) model [5]. This idea has been modified and implemented to predict SFPs using gene expression data profiled by Affymetrix GeneChip arrays in more complicated species such as *Arabidopsis* [6] and barley [7],[8].

On the basis of analyzing the Affymetrix microarray datasets of gene expression profiled at two developmental stages of two elite barley parental varieties and their 30 doubled-haploid segregation lines, Luo et al recently found that a large proportion of the SFPs predicted from the methods proposed in [4] and [1] were actually gene expression markers (GEM) [9]. Although the integration of genetic polymorphism detection and gene expression analysis undoubtedly has tremendous value in genomics studies, serious problems may arise if the SFPs so predicted are used as genetic markers in conventional QTL analysis or genetical genomics analysis [10]–[12]. It is an essential feature for genetic markers to be devoid of effect on the traits under question. In the scenario of expression QTL (eQTL) analysis, an autocorrelation between the expression level and GEM for a gene will always lead to inference of a *cis*-acting regulator for the gene even though the gene is actually *trans*-regulated.

In this paper, we present a novel method with improved robustness and accuracy for identifying and genotyping SFPs from Affymetrix gene expression data profiled on two parental lines (or strains) and their offspring population. We demonstrate the efficiency and robustness of the method by analyzing genomic DNA microarray hybridization profiles and RNA expression profiles of two laboratory strains of budding yeast (*S. cerevisiae*) and their 40 segregants as well as gene expression profiles at the embryonic stage of two elite barley varieties (Steptoe and Morex) and their 139 doubled haploid (DH) lines. The method was compared to those documented in both historical and more recent literature [1],[4],[6],[7],[8].

## Results

It has been theoretically demonstrated that hybridization intensity of a transcript molecule onto its target probe on an Affymetrix GeneChip oligonucleotide microarray can be modelled as a product of the binding affinity between the transcript and probe sequences and the abundance of the transcript [9]. This explains the multiplicative model for perfect match hybridization intensities, firstly proposed in [13]. The model confers two useful properties in the present context. Firstly, it enables separation of the binding affinity parameter from the transcript abundance parameter. The former reflects the degree of homogeneity between the transcript and its probe sequences. The latter is a function of the expression level of the gene represented by the probe set. Secondly, both parameters can be estimated from a properly designed experiment, thus avoiding the confounding influence of gene expression when comparing the binding affinity of the same probe between different genotypes. On the basis of this proposition, we developed a new method (Method **1**) for identifying and genotyping SFPs by making use of the estimated hybridization affinity from gene expression data profiled on two parental strains or lines and their segregating progeny with Affymetrix oligo-microarrays.

We explored the new method and compared it with five existing methods for predicting SFPs, Method **2** [1], Method **3** [4], Method **4** [7], Method **5** [6] and Method **6** [8]. Method **1** is explained in the Methods section, and the others outlined in the supplementary Text S1 available at the website of *PLoS Computational Biology*. We implemented all six methods in the Fortran-90 computer language to analyze three Affymetrix microarray datasets. The first two datasets were genomic DNA and cRNA microarray hybridization datasets collected from the same budding yeast population consisting of two parental strains, each of which was replicated three times, and their 40 haploid segregant offspring without replication. The third dataset was collected from a cRNA microarray hybridization experiment on two commercial varieties of barley, each of which was repeated three times, and their 139 doubled-haploid progeny lines without replication.

### Consistency in SFPs predicted from parallel DNA and RNA datasets

With gene expression data, one may anticipate a large variation in abundance of transcripts among different genes within the same individual or between different individual genotypes. However, with genomic DNA microarray data, one can expect uniformity in the number of DNA molecules hybridized onto a microarray chip across all genes interrogated on the chip. We first compared the SFPs from the two parallel yeast (DNA and RNA) microarray datasets by the six different methods. It can be seen from Table 1 that the present method (Method **1**) predicted a total number of 4107 (2077+2030) SFPs from the yeast DNA dataset and a total of 2388 (2077+311) from the RNA dataset. The number of SFPs called in both the datasets was 2077 by this method, indicating that 87% (2077/2388) of the SFPs called by the method in the RNA data were also detected in the DNA data. This percentage is the highest among the six methods. Because the SFPs called from RNA data, which were also recovered in DNA data, are more likely to be the true sequence polymorphisms, the present method thus confers the highest accuracy in identifying the SFPs bearing sequence polymorphisms from RNA microarray data. This also suggests that the present method could be implemented for predicting SFPs from the DNA dataset as well as from the RNA microarray dataset.

**Table 1:** The number of SFPs detected by the present and other methods from the yeast genomic DNA and RNA microarray datasets.

SFPs called	Method 1	Method 2	Method 3	Method 4	Method 5	Method 6
	(Present)	(Winzeler et al 1998)	(Ronald et al 2005)	(Cui et al 2005)	(West et al 2006)	(Rostoks et al 2005)
Shared^a^	2077	107	2475	429	1260	1401
Unique in DNA^b^	2030	3385	1550	574	2062	2697
Unique in RNA^c^	311	143	732	809	424	921
% shared^d^	87	42.8	77.1	34.7	74.8	60.3

a the SFPs called in both DNA and RNA microarray datasets.

b the SFPs called only in DNA microarray data.

c the SFPs called only in RNA microarray data.

d percentage of SFPs called in RNA data that were also called in the DNA.

All the methods except for method **4** predicted a larger number of SFPs from the DNA dataset than from the RNA dataset, as expected given the fact that RNA microarray data involves much larger variation than genomic DNA data. Among the six methods explored here, method **4** proposed in [7] shows the poorest performance, predicting the smallest number of SFPs in the two datasets and having the lowest consistency in the SFP prediction from the two parallel datasets. Method **2** was originally developed to predict SFPs from genomic DNA microarray data. Table 1 indicates that this method revealed a comparable number of SFPs from the DNA data to the numbers called by the other methods (except for Method **4**). However, its predictability was significantly worse in the RNA data analysis, reflecting a remarkable difference in recovering SFPs between the two types of Affymetrix microarray data.

### Efficiency in avoiding gene expression markers

It has been demonstrated by Brem et al that up to 43% of genes may express differentially between two laboratory yeast strains [14]. Differential gene expression between two parental strains has a high heritability with an average of 84%. These observations in yeast were also observed in the genomes of other more complex organisms [12],[15],[16],[9]. This heritable variation in gene expression was referred to as gene expression markers (GEM) by West et al [6]. It is essential to minimize the chance of calling a GEM in the SFP prediction for a robust genetical genomics analysis particularly when both genetic marker information and gene expression are extracted from the same RNA microarray dataset. Table 2 lists the numbers of SFPs and SFP-bearing genes, which carried at least one SFP, called by each of the six different methods as well as the proportion (in parentheses) of the SFP-bearing genes that were differentially expressed between the two parental lines in the yeast and barley RNA microarray experiments. A gene in these experiments was tested for differential expression between the two parental genotypes by fitting its expression level, which was evaluated from the Affymetrix recommended software MAS 5.0, if either of the genotypes exceeded the expression level of the other genotype by two fold or more. We explored use of SAM (Significance Analysis of Microarrays) proposed in [17] to test for significance of difference in gene expression between the two parental genotypes and found a very similar pattern to the MAS 5.0 analysis. Although Method **3** consistently detected the largest numbers of SFPs and SFP-bearing genes from both the RNA datasets (yeast and barley) among the six methods, the proportion of the differentially expressed genes called by this method was about two (in yeast data) to three (in barley data) times as many as that by Method **1** developed in the present study. The consistently lowest proportions of differentially expressed genes called by the present method in the two independent RNA microarray experiments demonstrated its effectiveness in avoiding GEMs in the SFP prediction. Method **6** was originally proposed to detect SFPs from Affymetrix microarray data from hybridizing RNA of multiple tissues of two genotypes [8]. The number of SFPs predicted from the method depends on the use of different stringency parameters (see [8],[17] for details). Although SFP predictions were made at a series of stringency parameters (supplementary Table S1), we chose the one at which the number of SFPs predicted was comparable to that by Method **1**. It is clear from Table 2 that Method **6** calls SFPs associated with differentially expressed genes much more frequently than the method developed in the present study.

**Table 2:** The numbers of SFPs and SFP-bearing genes called by the six different methods from the yeast and barley gene expression datasets.

Method	Species			
	Yeast		Barley	
	SFPs	Genes (%)	SFPs	Genes (%)
**1** (Present)	2388	1801 (5.6)	3206	2509 (6.7)
**2** (Winzeler et al 1998)	250	199 (9.0)	2105	1674 (8.4)
**3** (Ronald et al 2005)	3207	2280 (9.2)	4323	2869 (18.2)
**4** (Cui et al 2005)	1238	841 (11.7)	2368	1137 (28.1)
**5** (West et al 2006)	1684	1297 (8.0)	971	753 (23.4)
**6** (Rostoks et al 2005)	2322	1726 (8.2)	3294	2160 (21.2)

The percentage of genes differentially expressed between the two parental lines is given in parentheses.

### Mutual predictability among the six methods

One important aspect in comparing the different methods would be to compare their mutual predictability to the same SFPs. The figures listed in diagonal cells of Table 3 were the numbers of SFPs predicted from the yeast DNA data, yeast RNA data and the barley RNA data accordingly for the methods **1**–**6**. The upper and lower diagonal cells were the numbers and percentages (in parentheses) of the SFPs called by method **j** (*j* = 1^st^, 2^nd^, …, 7^th^ column) and also by Method **i** (*i* = 1^st^, 2^nd^, …, 7^th^ row, *i* ≠ *j*). For example, Method **1** predicted 1606 out of the 3492 SFPs predicted by Method **2** in the yeast DNA data. Thus, the predictability of Method **1** to Method **2** was 1606/3492 = 46%. Conversely, the predictability of Method **2** to Method **1** was 1606/4107 = 39% in the same dataset.

**Table 3:** Comparison in SFP predictability between methods 1–6 from the three datasets.

Methods	1	2	3	4	5	6
**1** (Present)	4107^a^	1606(46%)	3546(88%)	967(96%)	2963(89%)	2237(54%)
	2388^b^	61(24%)	2141(67%)	1040(84%)	1384(82%)	1665(71%)
	3206^c^	202(10%)	1704(39%)	1164(49%)	588(61%)	1688(51%)
**2** (Winzeler et al 1998)	1606(39%)	3492	1533(38%)	5(0%)	926(28%)	900(21%)
	61(3%)	250	88(3%)	7(1%)	30(2%)	20(0%)
	202(6%)	2105	261(6%)	120(5%)	31(3%)	129(3%)
**3** (Ronald et al 2005)	3546(86%)	1533(44%)	4025	934(93%)	3023(91%)	2260(55%)
	2141(90%)	88(35%)	3207	1129(91%)	1559(93%)	1955(84%)
	1704(53%)	261(12%)	4323	1102(47%)	497(51%)	1714(452%)
**4** (Cui et al 2005)	967(24%)	5(0%)	934(23%)	1003	979(29%)	730(17%)
	1040(44%)	7(3%)	1129(35%)	1238	946(56%)	1064(45%)
	1164(36%)	120(6%)	1102(25%)	2368	667(69%)	1638(49%)
**5** (West et al 2006)	2963(72%)	926(27%)	3023(75%)	979(98%)	3322	2044(49%)
	1384(58%)	30(12%)	1559(49%)	946(76%)	1684	1328(57%)
	588(18%)	31(1%)	497(11%)	667(28%)	971	752(22%)
**6** (Rostoks et al 2005)	2237(54%)	900(25%)	2260(56%)	730(72%)	2044(61%)	4098
	1665(69%)	20(8%)	1955(60%)	1064(85%)	1328(78%)	2322
	1688(52%)	129(6%)	1714(39%)	1628(68%)	752(77%)	3294

The upper and lower diagonal cells were the numbers and percentages (in parentheses) of the SFPs called by method **j** (*j* = 1^st^, 2^nd^, …, 7^th^ column) and also by Method **i** (*i* = 1^st^, 2^nd^, …, 7^th^ row, *i* ≠ *j*). For example, Method **1** predicted 1606 out of the 3492 SFPs, predicted by Method **2** in the yeast DNA data. Thus, the predictability of Method **1** to Method **2** was 1606/3492 = 46%. Conversely, the predictability of Method **2** to Method **1** was 1606/4107 = 39% in the same dataset.

a Yeast DNA data.

b Yeast RNA data.

c Barley RNA data.

It can be seen from this table that Method **1** predicted a high proportion of 54∼96% of SFPs called by Methods **3**–**6** in the two yeast (DNA and RNA) datasets. The proportion decreased to 39∼61% in the barley RNA data. Methods **1** and **3** predicted a very comparable and high proportion of the SFPs called by Methods **4** and **5**. However, the latter two methods predicted only a much smaller proportion of SFPs called by the former. This is partly a reflection that the numbers of SFPs predicted by Methods **1** and **3** are much larger than those by Methods **4** and **5** from all the three datasets. In the two RNA datasets, Method **1** predicted smaller proportions of SFPs called by Method **3** than the proportion predicted by Method **1** but also called by Method **3**. This reflects a larger number of SFPs called by Method **3** than by Method **1** because the former is prone to including GEMs in the SFPs called. If the comparison was made after removing those SFPs in the differentially expressed genes, the mutual predictability between the two methods becomes more comparable (47∼71% vs. 51∼90%).

### Efficiency to predict sequence polymorphisms

Sequence information was available for 10 genes of the two yeast parental strains. A BLAST analysis revealed that the sequences cover a total of 98 probes interrogated on Affymetrix yeast 2.0 Gene Chips. In six of the 98 probe sequences, the two yeast strains were polymorphic. There were 518 DNA segments sequenced for the two barley parental lines, which covered a total of 4690 probes on Affymetrix barley 1.0 chips. The two parental lines were polymorphic at 167 probes sequenced. It should be stressed that these sequence data were collected from other independent research projects conducted before the present study. From these sequence data, we calculated the probability (*s*) of a probe bearing genetic polymorphism given it is called as an SFP and the probability (*r*) of a probe bearing genetic polymorphism given it is not called as an SFP. The former reflects the true discovery rate of genetic polymorphism from predicted SFPs and the latter measures the rate of false negatives in the predicted SFPs. By definition, 1-*s* gives the rate of false positives.

Let *N* be the total number of probes interrogated on the yeast 2.0 or barley 1.0 Affymetrix microarray chip. Among *M* sequenced probes, there are *m* showing polymorphism between the two parental lines. For a given SFP prediction method under question here, *K* out of *n* predicted SFPs are included among the sequenced SFPs (*K*≤*M*) and *k* of the *K* SFPs are found polymorphic (*k*≤*m*). Based on the observed numbers, the most likely estimates of *s* and *r* can be calculated from 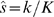 and 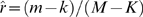.

Table 4 lists the observed numbers of the aforementioned parameters and the estimates of the parameters describing the true discovery rate and the rate of false negatives of SFPs predicted by the six different methods from the three microarray datasets. Method **1** recovered all 6 and 4 out of the 6 probes that carried genuine sequence polymorphism in the yeast DNA and RNA datasets respectively without making any false positive discoveries, showing that the method had an estimated true discovery rate of 100% and an estimated rate of zero false negatives in the SFPs predicted from the yeast DNA and RNA microarray datasets. In the barley RNA data, the method identified 57 SFPs out of 167 probes confirmed with sequence polymorphism, suggesting that 35% SFPs predicted by Method **1** bear genuine sequence polymorphism and 2% of polymorphism free probes were called as SFPs by the method. Although Method **1** performed much worse in recovering true polymorphic SFPs and avoiding false negatives in the predicted SFPs in the barley data than in the yeast datasets, it is clear that the method confers a better predictability of genuine sequence polymorphisms and a lower risk for calling polymorphism-free probes to be SFPs when compared the other five methods.

**Table 4:** The numbers of total probes interrogated on yeast 2.0 and barley 1.0 Affymetrix microarray chips (*N*), SFPs called (*n*), probes sequenced (*M*), probes bearing sequence polymorphism (*m*), probes sequenced and called as SFPs (*K*) and SFPs bearing sequence polymorphisms (*k*), and the estimates for rates of true discovery (*s*) and false negative (*r*).

Dataset	Method^a^	*N*	*n*	*M*	*m*	*K*	*k*	*s*	*r*
Yeast (DNA)	**1**	62810	4107	98	6	6	6	1.00	0.00
	**2**	62810	3492	98	6	3	2	0.67	0.04
	**3**	62810	4025	98	6	6	6	1.00	0.00
	**4**	62810	1003	98	6	3	2	0.67	0.04
	**5**	62810	3322	98	6	5	5	1.00	0.01
	**6**	62810	4098	98	6	9	5	0.56	0.01
Yeast (RNA)	**1**	62810	2388	98	6	4	4	1.00	0.02
	**2**	62810	250	98	6	0	0	0.00	0.06
	**3**	62810	3207	98	6	5	4	0.80	0.02
	**4**	62810	1238	98	6	5	4	0.80	0.02
	**5**	62810	1684	98	6	13	6	0.46	0.00
	**6**	62810	2322	98	6	7	4	0.57	0.02
Barley (RNA)	**1**	250811	3206	4690	167	163	57	0.35	0.02
	**2**	250811	2105	4690	167	120	19	0.16	0.03
	**3**	250811	4323	4690	167	192	44	0.23	0.03
	**4**	250811	2368	4690	167	139	43	0.31	0.03
	**5**	250811	971	4690	167	83	24	0.29	0.03
	**6**	250811	3294	4690	167	147	44	0.30	0.03

a **1** – The present; **2** – Winzeler et al 1998; **3** – Ronald et al 2005; **4** – Cui et al 2005; **5** – West et al 2006; **6** – Rostoks et al 2005.

It should be noted that the number of probes sequenced is very limited in comparison to the total number of probes. This indicates that the estimates of *s* and *r* may have large sampling variances, suggesting a larger number of probes need to be sequenced to provide more precise estimates of the parameters. However, as aforementioned, the sequenced probes represent an independently and arbitrarily chosen sample of all probes. Thus, comparative assessment of performance of the methods in predicting truly polymorphic SFPs should be informative. In addition, the number of SFPs predicted by one method varies markedly from that by other methods. Compared to Method **1**, Methods **2**, **4** and **5** predicted a much smaller number of SFPs. This may suggest that these methods may apply a more stringent selection criterion in prediction of SFPs. Nevertheless they did not produce more accurate prediction of true sequence polymorphisms in the predicted SFPs. Without influence of gene expression differentiation as in the yeast DNA microarray data, performance of Methods **1** and **3** is comparable. However, the former performed clearly better than the latter in the RNA, particularly in barley RNA data.

All six methods consistently performed considerably better in SFP prediction from the yeast than from the barley data.

### Genotyping and map construction with SFPs

Of the 139 DH lines in the barley RNA dataset, 30 were genotyped at the 518 single nucleotide polymorphic markers (SNPs) in another independent research project. We firstly explored Methods **1**–**5** for their ability to predict the genotypes of the 30 DH lines at the SFPs called by each of the methods and whose polymorphic status were confirmed by sequence information. Method **6** did not provide genotype prediction and was thus excluded from further analysis. Percentages of erroneously genotyped DH lines at different numbers (in parentheses) of truly polymorphic SFPs were 2.5 (57), 2.7 (19), 4.6 (44), 4.0 (43) and 2.0 (24) for each of the five methods accordingly. It is clear that the present method has a slightly higher (by 0.5%) genotyping error than Method **5**, but a lower genotyping error than the other methods. It should be noted that the erroneous genotyping rate reported for Method **5** was based on less than half the number of SFPs on which the present method was assessed.

**Figure 1:**
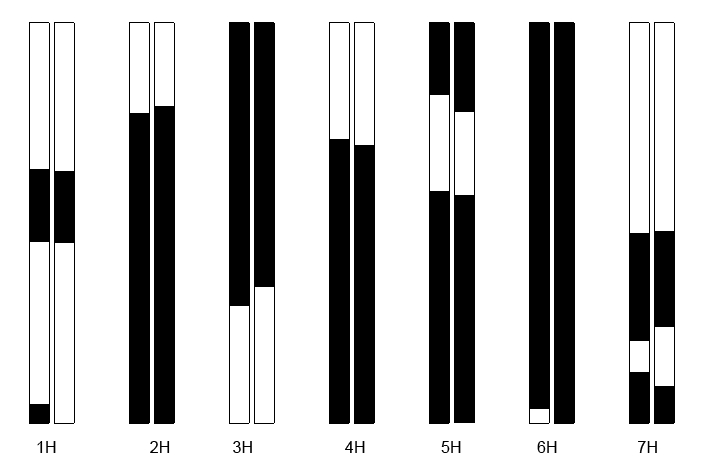
Haplotypes of chromosomes from line SM116 drawn to compare SFP (left) and SNP (right) predictions. Black bars = St chromosomes, white = Mx chromosomes.

Another useful test of the accuracy in genotyping the DH lines at a larger scale of SFPs predicted by the present method is the extent of agreement in the haplotypes and genetic linkage maps which are constructed from the genotype data on the SNPs and the SFPs predicted. We performed a post-genotyping filtering process to eliminate those SFPs with >10% missing genotypes scored, distorting from the 1∶1 segregation ratio and showing identical genotypes at any pair-wise loci. After this screening process, a total of 1381 SFP markers remained. JoinMap, a least-square based computer software [18], was employed to join the SFP markers into linkage maps. At LOD = 5.0, all but 3 SFP markers were clustered into 7 linkage groups, corresponding to the seven chromosomes of barley. We also compared chromosomal haplotypes constructed for each of the 30 DH lines from the SNP markers and from the SFPs. Figure 1 illustrates the 7 chromosomal haplotypes of the DH line, which bears the largest number of recombination events, constructed from the SFP (left) and SNP (right) markers. The good agreement between the two groups of chromosomal haplotypes suggests that genetic maps built using the SFP markers identified and genotyped from the present method would be as reliable as those constructed by using conventional DNA molecular markers. The 1,378 SFPs predicted in the present study were well mapped into seven linkage groups, corresponding to the seven barley chromosomes. The linkage maps were illustrated as Supplementary Figure S1. This probably represents the barley linkage maps with the densest currently available marker coverage.

## Discussion

Integrating identification of genetic polymorphisms and analysis of gene expression from a single experiment that hybridizes cRNA samples onto Affymetrix oligo-nucleotide microarrays has an extremely useful implication in at least two aspects. Firstly, it improves both accuracy and precision in calculating gene expression indices by excluding the probes involving genetic polymorphisms. Secondly, it enables generation of an abundant number of reliable genetic markers and, in turn, mapping genetic regulators controlling gene expression. Compared to modelling genomic DNA microarray data, modelling RNA data for this purpose raises remarkable analytical challenges because the effect of genetic polymorphism within a transcript molecule on hybridization signal of its target probe is always coupled with expression level of the gene represented by the probe. To minimize the influence of the confounding effect of gene expression has been the central topic for a robust diagnosis of the single feature polymorphisms (SFPs) from RNA microarray data [6],[7]. In this paper, we proposed to separate binding affinity between a transcript and its target probe from the abundance parameter of the transcript based on the multiplicative regression model described in [13]. The binding affinity parameter would reflect any sequence variation in the transcript sequence, while the abundance parameter is a measure of expression level for the gene.

We presented here a novel statistical approach for discovering and genotyping SFPs from oligonucleotide microarray expression data based on estimates of the binding affinity parameter. The approach (Method **1**) was compared to those which were designed for detecting SFPs in historical (Method **2**, [1]) and more recent (Method **3**–**6**, [4],[7],[6] and [8] accordingly) literature. We explored the methods from several angles. Firstly, their robustness to the influence of differential gene expression is assessed by comparing the SFPs predicted from parallel genomic DNA and mRNA hybridization datasets collected from the same set of two parental yeast strains and their 40 haploid offspring segregants. Because we can safely postulate a very high level of uniformity in the number of genomic DNA molecules hybridized onto their target probes across different arrays, a higher proportion of the SFPs predicted by a method from both the datasets must indicate greater robustness of the method to variation in gene expression. The present method yields a proportion of SFPs simultaneously detected from the two datasets which is at least 10% higher than each of the five other methods. In addition, we investigated the methods for their predicted SFPs in the genes that were differentially expressed in the yeast RNA dataset and another independent RNA dataset containing expression profiles from two commercial barley varieties and their 139 DH lines. Compared to its rivals, the present method calls the lowest proportion of SFPs involving differentially expressed genes. These observations clearly demonstrate that the SFPs called by the present method would be those more likely due to sequence variation than to differentiated gene expression. This is important for the SFPs to be used as genetic markers in mapping quantitative trait loci (QTL) or expression of genes (eQTL) because it is essential for any genetic markers to be devoid of any biological and functional effect. In particular, use of gene expression markers (GEMs) in eQTL analysis will inevitably result in false declaration of *cis*-transcriptional regulators resulting from autocorrelation between the GEMs and expression trait phenotype.

Lack of full sequence information at the SFPs predicted to be used as “gold standard” hinders a direct assessment for their polymorphic status in the previous studies. In this study, we evaluated the six methods for their ability to recover true sequence polymorphisms in the SFPs predicted based on sequence information for a limited number of probes (98 in the yeast experiment and 4690 in the barley experiment). With information of sequenced probes in both yeast and barley datasets, we are able to calculate the probability of a probe bearing genetic polymorphism given it is called as an SFP and the probability of a probe bearing genetic polymorphism given it is not called as an SFP for each of the six methods. These probabilities enable evaluation of the methods for the true discovery rate and the rates of false positive and false negative in recovering true genetic polymorphisms from the predicted SFPs. The present method shows the highest true discovery rate and the lowest rate of false negative across all three datasets. The method, although developed for SFP prediction from RNA microarray data, performed markedly better in unravelling the true sequence polymorphisms from DNA microarray data when compared with Method **2** [1], which was originally designed for SFP prediction from the same dataset. Methods **4** and **5** ([7],[6] respectively) predicted a considerably smaller number of SFPs when compared to Method **1** and this implies that these methods have applied a more stringent criterion in detecting the SFPs but this has not resulted in more accurate prediction of SFPs. The number of SFPs detected by Method **6** [8] is largely dependent on the prior use of stringency parameters, so we compared this method using the stringency criterion that leads to a similar number of SFPs predicted by the method to that by Method 1. One may argue that the improved performance of Method **1** in predicting truly polymorphic SFPs is due to the markedly larger number of SFPs predicted by the method. Given the fact that, among the sequenced probes, the number of probes without polymorphism is much larger than that of the probes with genetic polymorphism, Method **1** has consistently the lowest estimated probability to call the polymorphism-free SFPs in all three datasets, indicating that the present method outperforms the others, but this is not due to its larger number of predicted SFPs.

**Figure 2:**
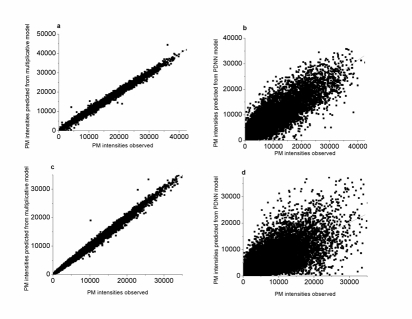
Regression analysis of predicted PM hybridization intensities from the multiplicative model (a and c) and PDNN model (b and d) with the yeast (a and b) and barley (c and d) data.

Methodologically, Methods **1** and **3** are developed on the multiplicative model described in equation (1) and the PDNN model [4] respectively for PM hybridization intensities from Affymetrix microarrays. We compared predictability of the two models by regressing the predicted PM intensities on the observed PM values from each of three replicates of the yeast parental strains and the barley parental lines. The regression coefficients of the predicted PM intensity from the multiplicative model on the observed PM intensity are 0.995∼0.998 (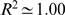) across the six yeast datasets and 0.994∼0.998 (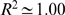) across the six barley datasets, but those from the PDNN model are 0.749∼0.788 (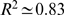) and 0.623∼0.658 (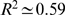) respectively. Figure 2 illustrates the correlation between the observed and predicted PM intensities from the present model (a, c) and PDNN (b, d) algorithm for the yeast (a, b) and barley (c, d) microarray datasets. It is clear that the multiplicative model shows remarkably better performance in predicting the PM intensities than the PDNN model. We also explored the predictability of the PDNN model in a logarithm-transformed scale and found that the logarithm transformation did not result in notable improvement in the predictability (data not shown but available upon request from the corresponding author). Obviously, an accurate prediction of the PM values constitutes an important basis for an accurate diagnosis of SFPs, and in turn, reliable genotyping of the SFPs. This at least partially, if not fully, explains why the two methods perform differently in the current context of application. It should be noted that this comparison does not necessarily indicate an evaluation of the methods' performance in calculating expression indices.

It should be highlighted that all six methods under investigation here consistently and considerably performed better in SFP prediction from the yeast microarray datasets (both DNA and RNA) than from the barley data. This difference could be largely explained by the fact that the yeast data were obtained from an advanced oligo-based microarray designed from information of the sequenced and well-annotated genome. In contrast, the barley gene expression data was obtained from the first generation Barley 1.0 Affymetrix microarray GeneChip assembled based on high quality expressed sequence tag information but without the benefit of whole genome sequence information. Design of the barley microarray probes could be less optimized than that of the yeast probes. Regardless of this limitation, Method **1** clearly outperformed its five rivals based on accuracy in detecting SFPs in the barley dataset.

When an SFP is identified between two individual strains or lines, assignment of a genotype at the SFP to each of them and their offspring, i.e. genotyping at the SFP, makes the second part of the SFP analysis. The present study, in which offspring lines or strains are not replicated, considered a much less demanding design of expression microarray experiments, in which offspring lines or strains are not replicated, than those in [6] and [7]. A practical challenge for the present method is that the binding affinity parameter can not be directly and independently estimated for each offspring individual without setting replications for these individuals in both the yeast and barley microarray experiments. To address this problem, we once proposed to approximate the probe binding affinity as simple functions of PM and MM hybridization intensities and calculated the probe effect independently for each of the offspring individuals (Equations 4.1–4.3 in [9]). The approximation may be questionable because it involves other effects in addition to the probe effect. Obviously, this biases estimates of the binding affinity parameter and thus could lead to poor prediction of the SFPs. Although there is insufficient data for directly estimating the binding affinity parameter at any given probe for each of the offspring, it is certain that each of the genes interrogated on the arrays must be from either of the two parental genotypes. On the basis of this observation, we developed a Bayesian approach to calculate the posterior probability of an offspring individual having inherited an allele from one of the two parents given the individual's and its parents' microarray data. We compared the genotypes predicted by the five different methods at the SFPs whose polymorphic status was confirmed by sequence information, for the 30 barley DH lines whose genotypes were known at 167 SFPs. At more than twice as many SFPs that contain confirmed sequence polymorphism, the present method correctly genotypes the DH lines with a rate very comparable to that by Method **5**, which has predicted the highest proportion of correct genotypes at the SFPs.

Our ability to extract information of genetic polymorphism from microarray gene expression data would be useful in at least the following aspects. Firstly, this enables integration of genetic marker development and gene expression profiling from only one set of RNA hybridization experimental data for recently launched gene expression QTL analyses. Secondly, the ability to genotype a population, while simultaneously measuring gene expression, is very valuable in a context where mislabelling and other quality assurance issues can easily occur. SFP genotype can be used to confirm the identity of individual (or sample) source material because the SFP genotype may be checked directly against previously obtained SNP genotypes. Thirdly, SFP prediction may allow allele-specific preferential gene expression to be explored when relative expression of a specific gene can be compared with that of its allele through detecting significance of the SFP associated effect on the gene expression. However, in either of these analyses, accuracy and robustness in SFP prediction is a crucial basis for their efficiency and reliability.

## Materials and Methods

### Yeast genomic DNA and RNA microarray hybridization experiment

YH1A, an isogenic haploid strain of the standard reference strain S288c, and YL1C, also a haploid strain that differed in ethanol tolerance from the former strain [19], were crossed to generate two backcross populations with each of the parental strains as recurrent parents. From each of the backcross populations, 20 offspring were randomly selected. This yielded 40 backcross strains. Total genomic DNA and mRNA were extracted individually from both the parental strains and the offspring strains. Then DNA and cRNA samples were labelled and hybridized to the Affymetrix Yeast Genome 2.0 GeneChip according to the supplier's guide manual (Affymetrix, GeneChip Expression Analysis technical manual 701021 Rev. 5. 2004). Each of the parental strains was repeated three times in both genomic DNA and RNA hybridization experiments, while the offspring were not repeated.

There were a total of 92 ( = 2×46) hybridized chips for yeast DNA and RNA datasets. Quality of the hybridization experiment was checked and confirmed by the standard method (GeneChip Expression Analysis Data Analysis Fundamentals; http:/www.affymetrix.com). We extracted hybridization data for 5,716 genes interrogated on the yeast 2.0 microarray chips and excluded 6 of them, which were represented by less than 11 probes. This resulted in a data set consisting of 62,810 ( = 5710×11) probe pairs present as a perfect match (PM) and a mismatch (MM).

### Barley RNA microarray experiment

Two commercial barley varieties, Steptoe (a feed variety)×Morex (a malting variety), were crossed to generate 150 doubled haploid (DH) lines. Preparation of plant material and embryo derived tissues for the microarray experiment can be found elsewhere [20]. RNA was isolated from the two parental lines and the 150 DH lines, processed and hybridized onto the Barley 1.0 Affymetrix microarray GeneChip. Technical details and protocols for running the microarray experiment can be found at www.biotech.iastate.edu/facilities/genechip/Genechip.htm. Each of the parental lines was repeated three times but the DH lines were not repeated in the microarray experiment. Of the 150 DH lines, 11 were removed for technical reasons. Thus, the barley RNA data analyzed in the present study was extracted from 145 (6+139) microarray chips. Altogether there were 22,801 different probe sets on every chip. Each probe set was represented by 11 pairs of perfect and mismatch hybridization values.

### Sources of sequence data

A total of 10 genes were partially sequenced for both yeast parental strains YH1A and YL1C. The sequence data had a total length of 16.6 kb. A BLAST analysis revealed that the sequence covered 98 probes on the Affymetrix yeast 2.0 chip. Partial DNA sequence information was available for a sample of 518 genes on the Affymetrix barley 1.0 chip for both parents and 30 of 139 DH lines. The sequence of 595.4 kb covered a total of 4690 probes on the barley chip. The sequence data was collected from other independent research projects conducted before the present study and used as “gold-standard” to assess accuracy of the six methods in identifying SFPs.

### Detecting SFPs

In an Affymetrix GeneChip, each gene is usually represented by a set of 11 probe pairs. Each of the probe pairs consists of a perfect match (PM) probe and a mismatch (MM) probe. The latter serves the role of distinguishing background noise from hybridization signal. Recent studies have shown that MM values do also detect hybridization signals [21]–[23], which raises the question about the reliability of estimating background noise from MM values. We thus consider PM values only in this analysis.

It has been shown that hybridization intensity of the *j*-th perfect match probe in the *i*-th probe set from an Affymetrix GeneChip can be modelled properly as a multiplicative model as given below
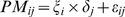
(1)
[13] and [9]. In equation (1), 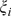 represents the model-based expression index of the probe set *i*, 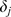 measures binding affinity between the transcript and probe *j* and 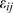 is a normally distributed residual variable of the model.

We consider a mating design in which two parental lines or strains are crossed to generate *n* offspring individuals. Genomic DNA or RNA samples are collected from the parents and offspring and hybridized to Affymetrix oligo-microarray chips. The hybridization experiment is repeated 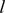 times for each of the parents, whose genotypes are denoted by *H* and *L* respectively, but done only once for each of the *n* offspring individuals. For simplicity but without loss of generality, the perfect-match hybridization signal may be represented by
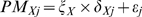
(2)with *X* = *H* or *L*. For each probe set, there are 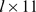 PM values observed on each of the two parental genotypes and they can be used to estimate the parameters in equation (2) through a restrained iterative least-square algorithm with the constraint 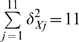 as proposed in [13]. For instance, let 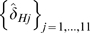 and 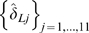 be the estimates of the 11 binding affinity parameters for the two parental genotypes respectively, then one calculates 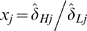 (*j* = 1, 2, …, 11). If the two parental genotypes are identical in the probe sequences, 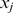 will be expected to take a value of 1.0 but deviate from the expectation if the *j*-th probe bears a polymorphic sequence between the two parents. To screen for the polymorphism bearing probe(s), i.e. single feature polymorphisms, we calculate the first (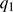) and the third (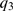) quartiles and inter-quartile range (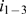) from 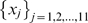. On the basis of the calculated quartiles, the *j*-th probe is inferred to be a candidate SFP if 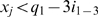 or 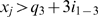.

### Genotyping SFPs

When an SFP is detected between the two parental lines, the next step is to determine the genotypes of their offspring at the SFP. The challenge in genotyping the offspring lies in that the offspring individuals are not replicated in the microarray experiment so that the binding affinity parameters can not be estimated independently for each of these individuals. Nevertheless, the SFP genotype for one of the individuals must be either *H* or *L*. Based on the argument, we develop here a Bayesian approach to calculate the probability that a particular individual has a genotype of *H* or *L* given its observed PM value and the distribution of the binding affinities of the two parental lines at the SFP.

We assume that binding affinity of each of the two parental genotypes (*X* = *H* and *L*) follows a normal distribution with mean and variance of 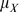 and 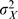 accordingly. Let 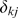 be the estimate of binding affinity of the *k*-th offspring individual at the SFP probe *j* and 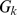 denote genotype of the individual at the probe *j*. The Bayesian probability has a form of
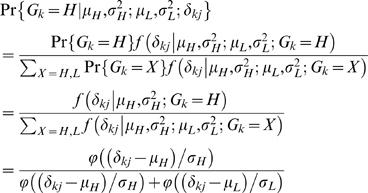
(3)because the offspring individual takes either of the parental genotypes with an equal probability of ½. In above equation, 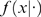 represents a conditional probability density function and 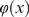 is the probability density function of the standard normal distribution.

We propose the use of the sample mean and variance as approximations of 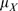 and 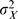 (*X* = *H* or *L*). For *m* hybridization replicates for each of the parents, there are a total of *m*(*m*-1)/2 possible pairs of PM values. Each of these paired PM values may be used to fit the model (1) and to generate an estimate of the binding affinity, 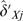. The sample mean and variance are then calculated from 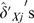. We denote the sample means and variances as 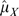 and 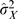. It should be noted that the variance may be underestimated because the data points are not completely independent each other. However, this may become negligible when the number of replicates, *m*, becomes large. To calculate the conditional mean 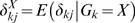 (*X* = *H* or *L*), we fit all *m* pairs of PM values (one from the conditional parent and the other from the offspring) to the model (1) and calculate the mean, 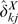, from the *m* estimates of binding affinity. On the basis of these estimates, we can numerically work out
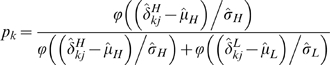
(4)and infer the offspring individual has genotype *H* if 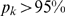 or genotype *L* if 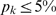, otherwise its genotype is uncertain.

### Computer programs and datasets

The programs developed to carry out the SFP diagnosis and genotyping presented in this paper are written in FORTRAN-90 and their executable versions with instructions are available upon request. We will make more user-friendly Windows-based applications in the longer term. The datasets analysed here are also available from the corresponding author.

## Supporting Information

Text S1 The supplementary text summarizes the key statistical algorithms developed by the five other methods under comparison in the present study for predicting single feature polymorphisms from Affymetrix microarray data.
(0.09 MB PDF)

Figure S1 Genetic linkage maps of 1,378 single feature polymorphism markers identified from two barley commercial varieties (Steptoe and Morex) and their 139 double haploid offspring.
(0.41 MB EPS)

Table S1 The number of SFPs called at different values of the stringency parameter delta by Method 6 (Rostoks et al 2005, Genome Biology 6, R54)
(0.03 MB XLS)
