# Supplementary material for: Robust Detection and Genotyping of Single Feature Polymorphisms from Gene Expression Data
Source: PLoS Comput Biol. 2009 Mar 13;5(3):e1000317. doi: 10.1371/journal.pcbi.1000317 (PMC2649212; doi:10.1371/journal.pcbi.1000317)
Supplement: Text S1 — The supplementary text summarizes the key statistical algorithms developed by the five other methods under comparison in the present study for predicting single feature polymorphisms from Affymetrix microarray data. (0.09 MB PDF) [file pcbi.1000317.s001.pdf]

Text S1

The supplementary text summarizes the key statistical algorithms developed by the five other methods under comparison in the present study for predicting single feature polymorphisms from Affymetrix microarray data.

Method 2: This was first proposed by Winzeler et al. (1998) to identify SFP from genomic DNA microarrays. Constancy in abundance of molecules interrogated for all genes is probably the most distinct feature of genomic DNA microarray data when compared to RNA microarray data. However, the two methods share a common principle in screening SFP, i.e. identification of the probes whose signal intensities contrast with the uniformity between the two genotypes for the remaining probes in the same set. The method would be appropriate to survey SFPs at least for those genes whose expression is not so different that the effect of the SFP associated probe will be hidden by variation in gene expression between the two genotypes. Thus, an obvious risk of using this method to predict SFPs is that genes differentially expressed between two genotypes are likely to be predicted as SFP associated genes even though there is no genetic polymorphism in the coding sequence of the genotypes. A detailed description of the method was summarized elsewhere (Brem et al. 2002) as follows

First, data from 3 hybridization experiments for each parent strain were filtered by removing probe pairs with saturated intensity (>40,000 units) on any chip, and probe pairs with a low perfect match (PM) intensity (<1,000 units) or a low difference between PM and mismatch (mm) intensities (<500 units) in any parent hybridization. All further analyses were performed on log(PM/MM) values.

To correct for global chip effect, each distribution was centered by subtracting its mode. We performed two scores for each probe pair:

$Z=D/(A+S)$ and $z=d/(a+s)$

Where

D is the average of 3 differences between two parental values (P_{1i}-P_{2i}, i=1,2,3), with P_{1i} being the ith value of the yeast parental strain (YH1A) or the barley parental

line (Morex).

S is their standard deviation, and

A is the 90th percentile of all S values

d is the average of the four differences P_{12}-P_{11}, P_{13}-P_{12}, P_{22}-P_{21} and P_{23}-P_{22}.

s is their standard variation, and

a is the 90th percentile of all s values.

We selected probe pairs with Z > 0.5. For all these selected probe pairs, we analyzed all hybridization experiments (parents and their offspring, 46 arrays for yeast data and 145 for barley data). We excluded probe pairs having missing values in more than 10 experiments, normalized log(PM/MM) values by dividing by the mode of their distribution, and for each probe pair, k-means clustering with k=2 was performed. Probes were retained if the clustering separated the parental values (i.e. all three P_1 values were clustered in one group and all three P_2 in the other). In the probes so selected, we calculated the probability of a given offspring individual having P_1 genotype as $P_1(x) / [P_1(x) + P_2(x)]$ in which $P_1(x)$ and $P_2(x)$ are the normal probability densities with the mean and variance calculated from P_1 and P_2 values respectively. If the probability >0.965 then the individual is inferred to have P_1 genotype or else if the probability <0.035 then the individual is inferred to have P_2 genotype or otherwise the individual's genotype is uncertain.

Method 3: Ronald et al (2005) developed an approach for predicting SFP and genotypes at the SFP in a yeast segregating population based on the proposition that the binding affinity of a transcript sequence to its complementary probe sequence can be adequately predicted from the positional-dependent-nearest-neighbour (PDNN) model (Zhang et al. 2003) as

$$\hat{I}_{ij} = N_i / \left[1 + \exp(E_{ij})\right] + N^* / \left[1 + \exp(E_{ij}^*)\right] + B \qquad (9)$$

For those species such as yeast considered by Ronald et al (2005), perfect-match probe sequences are known to exactly match their corresponding transcript sequences

in one of the parental strains from which the segregating population was created. In the standard strain, $\hat{I}_{ij}$ may be recognized as the expected value of perfect-match hybridization intensity of the jth probe for the ith gene. Under the PDNN model, N_i is defined as the expression index for the gene i and has a form of

$$N_i = \frac{\sum\left\{\left[I_{ij} - B - N^* / (1 + exp(E_{ij}^*))\right] / \lambda_{ij}\right\}}{\sum 1/[(1 + exp(E_{ij}))\lambda_{ij}]} \qquad (10)$$

where $\lambda_{ij} = \sqrt{I_{ij}[1 + exp(E_{ij})]}$ and I_{ij} is the observed perfect-match value. E_{ij} and E_{ij}^* are energy parameters depicting respectively specific and non-specific RNA-DNA binding and depend on nucleotide sequence of the target probe. Each of the energy parameters involves 40 unknown parameters (see Zhang et al. (2003) for details). Together with N^*, the non-specific binding parameter, and B, the constant background parameter, equations (9)-(10) involve a total of 82 unknown parameters to be estimated from $n \times 11$ perfect-match intensity values and probe sequences for each of the arrays in question by minimizing the so-called fitness function

$$F = \frac{1}{n \times 11} \sum_{i=1}^{n} \sum_{j=1}^{11} \left[log\hat{I}_{ij} - logI_{ij} \right]^2 \qquad (11)$$

where n is the number of genes interrogated. Ronald et al (2005) compared $I_{ij} / \hat{I}_{ij}$ of a yeast strain against that of the reference yeast strain. Significance of the comparison was taken as evidence to support inference of SFP associated with the probe.

Method 4: Cui et al. (2005) proposed an approach for predicting SFP and genotypes at the SFP in a barley segregating population based on estimate of probe affinity effect from a simple additive linear model of log-scaled perfect match signals. Let S_{tij} be the log-scaled PM value of the jth probe in the ith probe set hybridized to a RNA sample with genotype t (Here t = 1, 2 for the two parental genotypes). It was modeled

as

$$S_{tij} = I_{ti} + A_{tij} + \varepsilon_{tij} \tag{12}$$

where I represents the expression index at probe set level; A measures the probe affinity effect and ε is the residual term.

They denoted the difference of affinity effect between two genotypes by Y, a $N \times p$ matrix with elements

$$Y_{ij} = \text{sample median of } \widehat{A}_{1ij} - \text{sample median of } \widehat{A}_{2ij} \tag{13}$$

where i=1, 2, . . . , N and j=1, 2, . . . , p. More specifically, N row vectors of Y represent N distinct probe sets and p column vectors represent p probes which are tiled across a gene. Here, N row vectors are denoted by $y_1, y_2, \ldots, y_N$ for future use. The sample median was calculated on the biological replicates from each genotype.

Since it was expected that the majority of nucleotide positions within genes in the barley genome did not have polymorphism, parallel pattern of signal intensity between two genotypes should be observed in most probe sets. From a geometric point of view, if differentiation in signal intensity between two genotypes was represented by a p-dimensional point for each probe set, they would form a cloud in p-dimensional space with the majority of points clustered together and any point at the edge suggests a "potential" probe set that might contain SFP probe(s). Cui et al. (2006) used projection pursuit (Rousseeuw and Leroy 1987) to calculate overall outlying scores and individual outlying scores (defined below) to separate "potential" probe sets from the whole collection of probe sets under consideration and to quantify the contribution of individual probes to the overall outlyingness of their affiliated probe sets. Their algorithm can be summarized as follows:

(1) Fix a direction $\boldsymbol{v}$ (a $p \times 1$ vector). Project Y onto $\boldsymbol{v}$.

(2) Use relative absolute deviation to measure the outlyingness for every probe set on $\boldsymbol{v}$.

(3) Repeat steps (1) and (2) for all directions and take the supremum as the final overall outlying score for the probe set.

Let u_i be the *overall outlying score* for probe set y_i, i=1, 2, …, N. It is then

defined as

$$u_i = u_i(Y, \text{all}\,\boldsymbol{v}) = \sup_{\text{all}\ \boldsymbol{v}} \frac{\left|y_i'\boldsymbol{v} - med_j(y_j'\boldsymbol{v})\right|}{med_k \left|y_k'\boldsymbol{v} - med_j(y_j'\boldsymbol{v})\right|} \qquad (14)$$

where $y_j'\boldsymbol{v}$ is the usual inner product, i.e. $a'b = a_1b_1 + a_2b_2 + \ldots + a_pb_p$; med stands for the median (Rousseeuw and Leroy 1987). In practice, one cannot try all directions in the *p*-dimensional space since there are infinitely many. Cui et al. (2005) suggested using only those row vectors having high variation.

Next, a summary $\boldsymbol{w}_{ij}^*$ were proposed to evaluate the individual contribution (i.e. *individual outlying scores*) by each probe in a probe set:

$$w_{ij}^* = u_i(Y, \boldsymbol{v}_i^*) - u_i(Y \text{ with } Y_{ij} \text{ replaced by } med_j\{Y_{ij}\}, \boldsymbol{v}_i^*) \qquad (15)$$

with $\boldsymbol{v}_i^*$ being the particular direction at which u_i is actually obtained.

At last, the probe sets with the highest overall outlying scores will be identified as containing putative SFP probe(s). Then an SFP will be located at the probe with the highest individual outlying score. When multiple SFPs are involved, one can define the selection rule with certain stringency according to the real situation.

Method 5: West et al. (2006) suggested to identify SFP by calculating a summary measure, $SFPdev_i = |x_i - \bar{x}_{\notin i}| / x_i$, where x_i is the perfect match value of the *i*th probe in a given probe set and $\bar{x}_{\notin i}$ is the mean perfect match values of all remaining probes excluding the *i*th probe. West et al's algorithm was implemented by first searching for a bimodal distribution in the *SFPdev* values for all PM probes on the microarray from all the individuals. In brief, the *SFPdev* values were sorted into an ascending order $\{SFPdev_{j*}\}_{j*=1,n}$ with *n* being the number of individuals. A gap was determined at the *j**-th position when $(SFPdev_{j*+1} / SFPdev_{j*}) > 2.0$. Then an SFP was declared if the parental *SFPdev* values from the replicated GeneChips fell in separate ranges of the distributions. The $SFPdev_{j*+1}$ and $SFPdev_{j*}$ values for each putative SFP marker were

then used to define boundaries for assigning genotype score.

Method 6: Rostoks et al. (2005) presented a model-based approach for detecting SFPs. The model fits the logarithm of the background corrected and normalized perfect match signal, (Y_{ijkl}), for the *l*th replicate of the *k*th probe from the *j*th tissue of genotype *i* with a linear model

$$\log(Y_{ijkl}) = u + g_i + t_j + (g \times t)_{ij} + p_k + \varepsilon_{ijkl} \quad (16)$$

The residuals from this model were fitted for a genotype effect at the probe level to reveal SFPs using the Bioconductor package siggenes according to significance analysis of microarrays (SAM) (Tusher et al. 2001; Schwender et al 2003). A permutation test was carried out to calculate the false discovery rate of the significant genotype associated probe effect. Given a prior probability, say 0.95 as suggested, that a probe was not a SFP, a test statistic (delta) was calculated as distance between observed and expected likelihood of a probe being called an SFP and used to infer whether the probe under test was an SFP. Stringency of the statistical inference depends on the value of delta.

To implement the above approach to analyze the datasets in the present study, we used a simple model given as

$$\log(Y_{ijk}) = u + g_i + p_j + \varepsilon_{ijk} \quad (17)$$

for the background corrected and normalized perfect match signal of the *k*th replicate of the *j*th probe from genotype *i* because there was no tissue effect that needed to be modeled in the present datasets.

References

Brem, R.B., G. Yvert, R. Clinton, and L. Kruglyak. 2002. Genetic dissection of transcriptional

regulation in budding yeast. *Science* **296:** 752-755.

Cui, X.P., J. Xu, R. Asghar, P. Condamine, J.T. Svensson, S. Wanamaker, N. Stein, M. Roose, and T.J. Close. 2005. Detecting single-feature polymorphisms using oligonucleotide arrays and robustified projection pursuit. *Bioinformatics* **21:** 3852-3858.

Ronald, J., J.M. Akey, J. Whittle, E.N. Smith, G. Yvert, and L. Kruglyak. 2005. Simultaneous genotyping, gene-expression measurement, and detection of allele-specific expression with oligonucleotide arrays. *Genome Research* **15:** 284-291.

Rostoks, N., J.O. Borevitz, P.E. Hedley, J. Russell, S. Mudie, J. Morris, L. Cardle, D.F. Marshall and R. Waugh. 2005. Single-feature polymorphism discovery in the barley transcriptome.*Genome Biology* **6:** R54.

Rousseeuw, P.J. and A.M. Leroy. 1987. *Robust Regression and Outlier Detection.* John Wiley & Sons, New York.

Schwender, H., A. Krause, and K. Ickstadt, 2003. Comparison of the Empirical Bayes and the Significance Analysis of Microarrays. Technical Report. SFB 475: Dortmund .Germany: University of Dortmund.

Tusher, V. G., R. Tibshirani and G. Chu. 2001. Significance analysis of microarrays applied to the ionizing radiation response. *Proceedings of the National Academy of Sciences of the United States of America* **98:** 5116-5121.

West, M.A.L., H. van Leeuwen, A. Kozik, D.J. Kliebenstein, R.W. Doerge, D.A. St Clair, and R.W. Michelmore. 2006. High-density haplotyping with microarray-based expression and single feature polymorphism markers in Arabidopsis. *Genome Research* **16:** 787-795.

Winzeler, E.A., D.R. Richards, A.R. Conway, A.L. Goldstein, S. Kalman, M.J. McCullough, J.H. McCusker, D.A. Stevens, L. Wodicka, D.J. Lockhart, and R.W. Davis. 1998. Direct allelic variation scanning of the yeast genome. *Science* **281:** 1194-1197.

Zhang, L., M.F. Miles, and K.D. Aldape. 2003. A model of molecular interactions on short oligonucleotide microarrays. *Nature Biotechnology* **21:** 818-821.
